# Prevalence, antimicrobial susceptibility test and associated factors of Salmonella and Shigella in ready-to-eat fruit juices and salads in Mekelle, northern Ethiopia

**DOI:** 10.1186/s12879-024-09066-w

**Published:** 2024-02-13

**Authors:** Tesfay Berihu, Guesh Gebremariam, Yemane Weldu, Amlsha Kahsay, Tsehaye Asmelash, Araya Gebreyesus

**Affiliations:** 1 Department of Medical Laboratory Science, College of Health Science and Comprehensive Specialized Hospital, Aksum University, Axum, Tigray Ethiopia; 2 Department of Medical Microbiology and Immunology, College of Health Science and Comprehensive Specialized Hospital, Mekelle University, Mekelle, Tigray Ethiopia

**Keywords:** Antimicrobial susceptibility, Fruit juice, Salad, Salmonella, Shigella

## Abstract

**Background:**

Food borne diseases is a challenging problem nowadays. *Salmonella* and *Shigella* species are great concern of food-born outbreaks. Thus, this study was aimed to assess the prevalence, antimicrobial susceptibility test and associated factors of *Salmonella* and *Shigella* species in fruit juices and salads.

**Methods:**

A community based cross sectional study design was carried out on 50 juice houses from December to March 2020 in Mekelle. One hundred fifty samples were collected aseptically from the juice houses for laboratory analysis. Information related to risk factors was obtained using a structured questionnaire. In the laboratory, samples were homogenized using peptone water and incubated overnight for enrichment. Then, *Salmonella* and *Shigella* species were isolated on Salmonella-Shigella agar and Xylose Lysine Deoxycholate agar. Disc diffusion method was used to perform antimicrobial susceptibility test. Using SPSS (version 22) package, descriptive statistics and Chi square test (χ2) were used to analyze the data, and *p* < 0.05 was considered as statistically significant.

**Result:**

The overall prevalence of *Salmonella* and *Shigella* species was 41/150 (27.33%; 95% CI: 20.20 – 34.46) with 33 (22%) *Salmonella* spp. and 8(5.33%) *Shigella* spp. Antimicrobial susceptibility tests of both *Salmonella* and *Shigella* spp.showed high resistance against ampicillin (100%), tetracycline (63.6 and 62.5%, respectively) and amoxicillin-clavulanic acid (100%). Accessibility of fruits to flies and dust had statistical association (*p* = 0.021) with occurrence of *Salmonell a *and/or *Shigella* spp.

**Conclusion:**

The overall prevalence of *Salmonella* and *Shigella *spp. was found to be significant. The resistant rate of isolates against ampicillin, tetracycline and amoxicillin-clavulanic acid was high. Storage sites for fruits should be inaccessible to flies and dust. Therefore, routine monitoring of juice houses should be promoted and regular evaluation of bacterial resistance pattern should be done for selective antimicrobial therapy. Furthermore, consistent training of juice makers on food safety and hygiene should be implemented by the concerned body.

## Background

Nowadays, consumption of fruits and vegetables is increasing worldwide [1]. In developing countries, especially in large cities,a great number of people consume freshly squeezed fruit juices and salads [2]. Fruit juices are viscous liquids produced mostly from same or different edible fruits [3]. They are nutritious foods with favorable flavor and health benefits [4]. Consumption of freshly squeezed fruit juices provides great health benefits to humans. They are good sources of minerals, fiber,vitamins, and other substances [5].They provide essential antioxidants that are important in diabetes, cancer and heart diseases prevention. They also provide important nutrients that help for growing of acid tolerant bacteria [6].

Salad is a term used to refer to many food preparations of chopped or sliced mixtures, mainly vegetables or fruits [7].Salads are nutritious foods for human. They are highly important protective foods that are beneficial for the maintenance of health and the protection of diseases. They also contain valuable nutrients that are essential for the proper function of the body. Vegetables contain various medicinal and therapeutic agents used for the treatment of different diseases. They are also good sources of vitamins, minerals and other nutrients [8].

In spite of all the above advantages, food related infections are challenging problem due to climate change, food market globalization, and shifting human’sbehavior for consumption of fresh foods [9]. some researchers state that salads and fruit juices have been associated with outbreaks of food-borne diseases [10, 11].These foods are exposed to bacterial contamination from dust, soil, water, handling during harvesting and processing. Food-borne infections affecting the gastro-intestinal tract are mainly transmitted via the consumption of foods or drinks that are exposed to pathogenic microorganisms. The majority of microorganisms that are transmitted through food items are gram negative bacteria [12], of which *Salmonella* and *Shigella* spp. are great concern of food-bornepidemics [9].

Shigellosis and *salmonellosis* are highly infectious diseases of worldwide significance. Salmonellosis causes remarkable losses to society in most of the countries around the world [13].Globally, overa billion of diarrhealdiseases occur owing to salmonellosis yearly, resulting in deaths of 3 million. Moreover,indeveloping countries *Shigella* spp. causes more than 200 million cases and 650,000 deaths annually primarily among children and young adults [14]. Antimicrobial resistant bacteria/gene can contaminate foods in many ways. Antimicrobial resistant bacteria may be found in the soil, in the water and in human or animal fecal matter. Plant products may also be contaminated with antimicrobial resistant bacteria during production following the use of irrigation water contaminated with human and animal feces. Food borne drug resistant *Salmonella* spp. and *shigella* spp. are among the common pathogen for human health. The emerging resistance of these food borne pathogens results in rising in the number of in and out patient hospitalization and mortality rate [15].

As a developing country, Ethiopia is continually suffering from Salmonellosis and shigellosis [16].Moreover, drug resistance of *Salmonella* and *Shigella* species were reported to be increasing in Ethiopia. However, the source from which these infections are acquired is not extensively studied [17, 18].The consumption of fresh foods in Ethiopia is currently increasing [18].But information on the safety and quality of these foods prepared and consumed in Ethiopia is scanty and only a limited number of published documents exist. Particularly in Mekelle, there is no documented data in microbiological safety of most commonly consumed fruit juices (particularly mango juice and avocado juice) and salads. Therefore, this research was conducted to determine the prevalence, antimicrobial susceptibility test and associated factors of *Salmonella* and *Shigella *species in salads and fruit juices in Mekelle, Ethiopia.

## Materials and methods

### Study design, period and area

A community based cross sectional study design was done from December to March 2020 in randomly selected juice houses in Mekelle City, Ethiopia. Mekelle is the capital city of Tigray Regional State and is found at a distance of 783 km from Addis Ababa. It is located between 39^0^29′East longitude and 13^0^30^′^North latitude, with an elevation of 2084 m above sea level. The climatic conditions of Mekelle City conform to those of highlands [19].

Mekelle has a total of seven sub-cities, namely: Kedamay Weyane, Hawelti, Quiha, Hadnet, Semen, Adi Haki, and Ayder. Currently, the city has approximately 596 juice houses (191 in Kedamay Weyane, 81 in Hawelti, 12 in Quiha, 96 in Hadnet, 82 in Semen, 84 in Adihaki and 50 in Ayder [20].

### Sample size calculation

The sample size was determined using the following formula

$$\textrm{n}=\frac{Z^2\alpha /2\textrm{P}\left(1-\textrm{P}\right)}{{\textrm{d}}^2}$$

Prevalence of *Salmonella* spp. and *Shigella Spp* in food was taken as 9.1% from Ethiopia, Hawasa which was done by Eromo T.et al. [18]. Then with margin of error (5%), (d = 0.05) and 95% level of confidence (z = 1.96) the sample size was calculated as follows:

- n- Sample size
- P- Prevalence rate of *Salmonella* Spp. and *Shigella* Spp. from food (9.1%)
- Z- Confidence interval; by using 95% =1.96.
- D- is the margin of error, here we use margin of error 0.05.

$$\textrm{n}=\frac{(1.96)^{2\ast }0.091\ \left(1\hbox{-} 0.091\right)}{(0.05)^2}=127\textrm{plus}\ \textrm{contingency}\ 18\%\textrm{so},127+23=150$$

Therefore, a total of 150 samples from the seven sub-cities was taken.

### Sampling technique

According to the guidelines for sampling food microbiology [21], 50 juice houses were selected using clustered random sampling techniques from the seven sub cities and a total of 150 representative sample units (ready-to-eat avocado juices, mango juices, and salads) were taken from these juice houses. The number of juice houses to be sampled was allocated to each sub-city proportionally according to the number of juice houses using the proportionate allocation formula. Finally, three representative samples (avocado juice, mango juice, and salads) were taken from each randomly selected juice house.

### Data and sample collection, handling, transportation and processing

Structured questionnaire, direct interview and observation were used to obtain information such associo-demographic characteristic, hygienic status, sources and storage of fruits and vegetables from juice makers related to risk factors that may jeopardize the bacteriological quality and safety of the foods. Then, fresh ready-to-eat avocado juice, mango juice, and salad samples were purchased and collected in sterile containers directly from preparation rooms in the juice houses. Samples were labeled and placed into an icebox to maintain freshness and transported within 1-2 hours to the laboratory of Medical microbiology, College of Health Science, Mekelle University. From each collected juice sample, 25 g were homogenized in 225 ml of buffered peptone water and shaken vigorously. In the same way physiological saline was added to the collected salad samples and were shaken to dislodge adhered bacteria, 25 g of the shaken samples were transferred to 225 ml of buffered peptone water and properly mixed. All the homogenized samples were incubated at 37 °C for 24 hours for enrichment. After 24 hours of incubation, the enriched samples were used for the isolation of Salmonella and Shigella, and for serial dilutions to determine fecal coliforms [21].

### Microbiological analysis

#### Fecal coliform count

A three-tube most probable number (MPN) method was used to determine fecal coliforms. From the enriched samples, 1 ml was transferred into three test tubes containing double strength MacConkey broth (Oxiod, Hampshier, UK) and an inverted Durham’s tube. Then, from these test tubes, 1 ml of aliquot was aseptically transferred into a series of another 3 test tubes containing double strength MacConkey broth (Oxiod, Hampshier, UK) and Durham’s tubes. This continued up to three dilution series. Test tubes were shaken gently and incubated at 44.5 °C for 24 hours in water bath, and the then lactose fermentation and gas production were considered as a positive. To confirm the presence of *E. coli*, streaking on eosin methylene blue (EMB) (Oxiod, Hampshier, UK) plates and additional biochemical tests were performed. Finally, three tube MPN table was used to estimate bacterial loads [21].

#### Cultivation and identification of Salmonella and Shigella

After 24 hours of enrichment, samples were streaked on Salmonella Shigella Agar (Oxiod, Hampshier, UK) and Xylone Lysine Deoxycholate Agar (Oxiod, Hampshier, UK) and incubated at 37 °C overnight. After the overnight incubation,the presences of discrete colonies were screened. Then suspected colonies were subcultured on MacConkey Agar (Oxiod, Hampshier, UK) to get a pure colony. Finally, a single colony of bacterial isolates was taken for further identification by colony characteristics, Gram’s stain, and biochemical tests (H_2_S production,motility, carbohydrate fermentation, citrate utilization, gas production, indole production, and urease tests) using standard procedures [22].

### Antimicrobial susceptibility test

Modified Kirby-Bauer disc diffusion method was used perform antimicrobial susceptibility test according to Clinical and Laboratory Standards Institute (CLSI) guidelines [22, 23]. Antibioticslike ampicillin (AMP; 10 μg), amoxicillin/clavulanic acid (AMC; 20/10 μg), ceftriaxone (CTR; 30 μg), chloramphenicol (C; 30 μg), co-trimozaxole (COT; 25/125 μg), gentamicin (GEN; 10 μg), nalidixic acid (NA; 30 μg), ciprofloxacin (CIP; 5 μg) and tetracycline (TE; 30 μg) were used. These antibiotics were selected based on their frequent prescription and availability in the study area. According to the standardized table supplied by CLSI, isolates were classified as resistant, intermediate, and sensitive [23].

### Quality control

The legibility of the filled-out questionnaire was confirmed immediately. Samples were aseptically collected from the selected sites in sterile containers. They were labeled and placed in an icebox to maintain freshness and transported within 1-2 hours to laboratory of Medical Microbiology. Laboratory analysis was done using standard operating procedures (SOP) [22]. Culture media were tested for sterility and performance by incubating 5% of the batch [24]. Moreover, reagents were checked for expiry dates and proper functioning. During culture and antimicrobial susceptibility testing, standard reference strains of *S. enterica* (ATCC-29934) and *S. sonnei* (ATCC-25931) were used [22].

### Data processing and analysis

The collected questionnaires were checked for completeness and data analysiswere performed using Statistical Package for Social Sciences (SPSS) version 22 after the data cleaned and entered into a computer. Descriptive statistics of different variables were calculated and presented in the form of texts, tables and graph. Chi square test (χ2) was also done to examine the association between the dependent (prevalence of bacterial isolates) and independent variables (age, gender, educational status, training, storage sites, accessibility of vegetables to flies and dust, sources of fruits and vegetables, washing of fruits and vegetables, washing of equipment, awareness to microorganism, governmental sanitary regulation, and waste and garbage disposal), and a *p*-value ≤0.05 was considered as statistically significant.

## Results

### Socio-demographic characteristics of juice makers and other related data

Fifty juice makers were participated in this study. All participants were female. Three-fourth of them were aged less than 35 years. Majority (62%) of the respondents had educational status of high school and above. Only 1/4th of the juice makers were trained about food processing, hygiene and safety. During work,74% of the juice makers uses an apron. Respondents (100%) described that fruits and vegetables were brought from the open market retailers routinely. Most of the participants use shelves as temporary storage sites of fruits and baskets for storing vegetables (Table 1).

**Table 1:** Demographic Characteristics of Juice Makers (*n* = 50) and other Related Data on Juice Houses of Mekelle City, Northern Ethiopia (December-March 2020)

Variables	Response	Frequency	%
**Age**	Below 35 years 35 and above years	37 13	74 26
**Gender**	Female Male	50 0	100 0
**Education Status of Juice Makers**	Non-formal education Elementary High school and above	4 15 31	8 30 62
**Training on Food Hygiene and Safety**	Yes No	13 37	26 74
**Temporary Storage Sites of Fruits**	Shelf Basket Refrigerator	39 8 3	78 16 6
**Temporary Storage Sites of Vegetables**	Basket Refrigerator	36 14	72 28
**Accessibility of Fruits to Flies and Dust**	Yes No	38 12	76 24
**Accessibility of Vegetables to Flies and dust**	Yes No	0 50	0 100
**Use of Hair Restraint** **During Work**	Yes No	29 21	58 42
**Use of Apron During Work**	Yes No	37 13	74 26
**Washing of Equipments/ Utensils**	**-**Water only -Water and soap -Water, soap, bleaching agents	5 26 19	10 52 38
**Washing of Fruits and Vegetables**	Yes No	50 0	100 0
**Source of Fruits and Vegetables**	Directly from producers Open Market	0 50	0 100
**Awareness that Microorganisms can Contaminate Juices and Salads**	Yes No	41 9	82 18
**Sanitary Regulation from Government**	Yes No	50 0	100 0
**Waste and Garbage Disposal**	On the side of juice house Taken to remote waste disposal area	0 50	0 100

### Prevalence of Salmonella spp., Shigella spp. and Fecal coliforms loads among food items

**Fig. 1:**
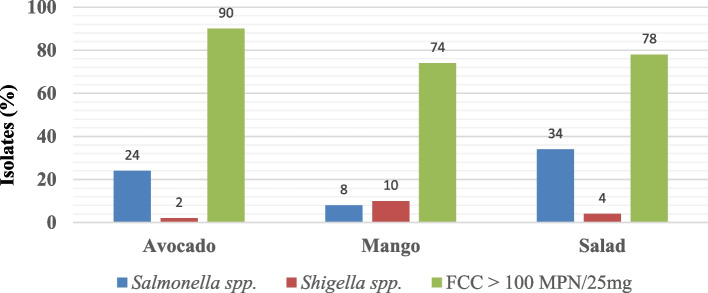
Prevalence of *Salmonella* spp., *Shigella* spp.*,* and Fecal Coliforms Loads (MPN/25 g) in Food items

Fecal coliform counts> 100 MPN/25g were shown in 90% of avocado juices, 74% of mango juices and 78% of salads (Fig. 1). Further biochemical tests indicated that all the fecal coliforms were detected to be *E.coli*.

### Prevalence of Salmonella and Shigella among sub-cities

Out of 50 juice houses examined, 27 (54%) had salmonella and/or shigella contamination. The majority of the positive juice houses were from: Kedamay woyane (18%), Adi-haki (10%), Hawelti (8%) and Hadnet (8%). Three juice houses had S*almonella* and/or S*higella* spp. contamination in all the three examined foods samples (avocado, mango and salads); 8 juice houses had contamination bacteria in two of the examined food samples; where as 16 juice houses had contamination of these bacteria in one of the samples examined (Table 2).

**Table 2:** Prevalence of Salmonella and Shigella spp.in Juice Houses in Different Sub-Cities of Mekelle,Northern Ethiopia (December-March 2020)

Sampling Site	Juice Houses Examined	Salmonella and/or Shigella Isolates	
		Yes n (%)	No n (%)
Kedamay Woyane	16	9 (18)	7 (14)
Hawelti	7	4 (8)	3 (6)
Hadnet	8	4 (8)	4 (8)
Semen	7	3 (6)	4 (8)
Adihaki	7	5 (10)	2 (4)
Ayder	4	2 (4)	2 (4)
Kuiha	1	0 (0)	1 (2)
**Total**	**50**	**27 (54%)**	**23 (46%)**

Out of 150 examined samples taken from 50 juice houses, 33 (22%) salmonella and 8 (5.33%) shigella were detected with an over all prevalence of 41 (27.33%; 95% CI: 20.20 – 34.46). Salmonella was dominant 17(34%) in salad, where as Shigella was slightly higher (10%) in mango juice. The occurrence of salmonella and shigella was higher in salads 19 (38%) followed by avocado juice 13 (26%) (Table 2).

### Antimicrobial susceptibility patterns

In this study, Salmonella and Shigella isolates (100%) were resistant against amoxacillin-clavulinic acidand ampicillin, where as gentamicin,ceftriaxone, ciprofloxacin, and chloramphenicol were potent for the isolates. Moreover, the resistance rates of the isolates against tetracycline were 78.78 and 75% respectively (Table 3).

**Table 3:** Antimicrobial Susceptibility Patterns of *Salmonella* spp. (*n* = 33) and *Shigella* spp. (*n* = 8) in Avocado Juice, Mango Juice and Salads in Mekelle, Northern Ethiopia (December-March 2020)

Antimicrobials	Bacteria isolates					
	***Salmonella*** spp. (***N*** = 33) n (%)			***Shigella*** spp. (N = 8) n (%)		
	R	I	S	R	I	S
Ciprofloxacin (5 μg)	0 (0.0)	0 (0.0)	33 (100)	0 (0.0)	0 (0.0)	8 (100)
Ceftriaxone (30 μg)	0 (0.0)	6 (18.2)	27 (81.8)	0 (0.0)	1 (12.5)	7 (87.5)
Gentamicin (10 μg)	0 (0.0)	1 (3.0)	32 (97.0)	0 (0.0)	0 (0.0)	8 (100)
Cotrimoxazole (25 μg)	2 (6.1)	1 (3.0)	30 (90.9)	0 (0.0)	2 (25.0)	6 (75.0)
Amox-clavul acid (30 μg)	33 (100)	0 (0.0)	0 (0.0)	8 (100)	0 (0.0)	0 (0.0)
Ampicillin (10 μg)	33 (100)	0 (0.0)	0 (0.0)	8 (100)	0 (0.0)	0 (0.0)
Nalidixic acid (30 μg)	6 (18.0)	10 (30.3)	17 (51.5)	0 (0.0)	3 (37.5)	5 (62.5)
Chloramphenicol (30 μg)	1 (3.0)	2 (6.1)	30 (90.9)	0 (0.0)	1 (12.5)	7 (87.5)
Tetracycline (25 μg)	21 (63.6)	5 (15.2)	7 (21.2)	5 (62.5)	1 (12.5)	2 (25.0)

*R* Resistant,  *I*  Intermediate, *S* Sensitive

### Multidrug resistance profile of isolates

Out of the 33 salmonella isolated, only 1 (3.0%) which was isolated from salad showed multidrug resistance, but none of the shigella species showed multidrug resistance (Table 4).

**Table 4:** Multidrug Resistance Patterns of Salmonella and Shigella Isolates in Ready-to-Eat Avocado Juice, Mango Juice,and Salads in Mekelle City, North Ethiopia (December-March 2020)

Bacteria isolates	Number of isolates	MDR^a^ isolates, N (%)
*Salmonella* spp.	33	1 (3.0)
*Shigella* spp.	8	0 (0.0)
**Total**	**41**	**1 (2.4)**

^a^Multi-drug resistant: non-susceptible to ≥ 1 agent in ≥ 3 antimicrobial categories

### Factors associated with the prevalence of Salmonella and Shigella

In the Chi square test (χ2), accessibility of fruits to flies (*P* = 0.021) was statistically associated to the occurrence of salmonella species and/or shigella species (Table 5).

**Table 5:** Factors Associated with the Prevalence of Salmonella and/or Shigella Isolates in Avocado Juice, Mango Juice and Salads in Mekelle city, Northern Ethiopia (December-March 2020)

Variables	Bacteria Occurrence		*P*-Value
	Yes	No	
Age Below 35 years	21	16	0.191
35 and above	6	7	
Educational Status			
Non-formal education	2	2	0.392
Elementary	7	8	
High school and above	18	13	
Awareness to Micro-organisms			0.525
Yes	22	19	
No	5	4	
Training on Food Safety and Hygiene			
Yes	3	10	0.990
No	24	13	
Storage Sites of Fruits			
Shelf	25	14	0.884
Basket	2	6	
Refrigerator	1	2	
Storage Sites of Vegetables			
Basket	23	13	0.781
Refrigerator	4	10	
Accessibility of Fruits to Flies and Dust			0.021^a^
Yes	24	14	
No	3	9	
Use of Hair Restraints			0.845
Yes	13	16	
No	14	7	
Use of Apron During Work			0.191
Yes	18	19	
No	9	4	
Washing of Equipments			0.759
Water only	3	2	
Water and soap	15	11	
Water, soap, bleaching	9	10	

^a^ Statistically significant with the occurrence of Salmonella and/or Shigella Isolates

## Discussion

The prevalence of *Salmonella* spp. (22%) the study was slightly similar with previously done studies in: Ethiopia, Bahir-Dar (20%) [25] and India (19.04%) [26], but higher than other reported findings in: Ethiopia, Hawassa (9.1%) [18] and Addis Ababa (10%) [27]; India (14.3%) [28]; Bangladesh (0.37%) [13]. However, our result was lower than earlier conducted studies in: Ethiopia, Bahir-Dar (57.5%) [29]; and India (36.55%) [2] and (33.8%) [30]. Moreover, a prevalence of 5.33% *Shigella* spp. was reported in the current study. This agrees with previous result in India (7.52%) [2]. But lower than the findings conducted in: Ethiopia, Addis Ababa (30%) [27]; and India (17.14%) [30].The possible explanation for this observable differences in the prevalence of these bacteria might be due to difference in hygienic practice [31]. Poor handling could also be a contributing factor to the variations in the bacteriological load [32]. Furthermore, the sources and storage period of the fruits and vegetables [33], seasonal variation [11]; and diversity of the pathogen in the environments might influence the results [18]. Transportation and storage, manipulation of temperature and storage period could also contribute to the multiplication of bacteria [33].

Salads and fruit juices are usually eaten raw or blended with other ingredients without heat treatment [34]. Fresh foods must be void from *Salmonella* and *Shigella* spp. [35]. It is unacceptable for consumption if *Salmonella and Shigella *spp. are present in 25 g of foods [36]. Therefore, contracting *Salmonella* and *Shigella* spp. in these food items is more vibrant [34]. Our study revealed that 38% of salad samples, 26% of avocado juice and 18% mango juice were found to be potentially hazardous to consumers, and needs serious public health concern. Potential high risk people are those individuals with waning immunity like immune-compromised, pregnant, young, and old aged [13].

The presence of high levels of fecal coliform indicates direct or indirect contamination from human and/or animal fecal origin. For ready-to-eat fresh salads and fruit juices, the maximum level of fecal coliforms permitted is 100MPN/25 g [35]. In the current study, 45 (90%) avocado juices, 37 (74%) mango juices and 39 (78%) salads had fecal coliform count > 100 MPN/25 g. Overall 121 (80.67%) of the examined food items were unsatisfactory for consumption. All salmonella and shigella isolates of this study were from the samples that had high fecal coliform count (> 100MPN/25 g). This suggests that these pathogens may originate directly or indirectly from human and/or animal (for *Salmonella* spp.) feces as a result of poor hygienic practice. Though occurrence of high number of fecal coliforms is not always associated with presence of pathogens, it may reflect poor hygienic quality of the foods and an increased likelihood presence of the pathogens. This contamination might be due to preparation of foods in unhygienic environment, and improper food handling [37].

In this study, Salmonella isolates showed 100% resistancerate against ampicillin. This was inline with the studies done in Ethiopia (100%) [38] and Malaysia (100%) [1]. Moreover, high resistance rate of *Salmonella* species (78.8%) against tetracycline in this finding was consistent with the former result reportedin Ethiopia, Jimma (100%) [39]. This high tetracycline resistant isolates may be due to the fact that this drug is one of the widely used for the treatment of infection in human as well as in livestock production [1]. The relative high rate of resistance against tetracycline, amoxicillin-clavulanic acid and ampicillinin this study indicates these agents are highly used in the environment [40]. An increase in the prevalence of bacterial strains resistant to antibiotic might be due to frequent use of antimicrobial in different fields [41]. Such high resistance properties against the commonly prescribed drugs may render these pathogens responsible for strict public health problems because of ineffective treatment of the sufferers [42].

This study also showed that antimicrobials like: ciprofloxacin (100%), ceftriaxone (81.8%), gentamicin (97%), co-trimoxazole and chloramphenicol (90.9% each) were effective against most salmonella and shigella isolates. Several previously conducted studies had similar result, such those in: Ethiopia [18, 29] and India [28], Thailand [43], Malaysia [1] and Pakistan [44] had comparable results. Low resistance of these bacteria against chloramphenicol in this study was also supported by a findings reported in Malaysia [45] which suggested that the low resistance rate of this drug was because of its restricted use in certain countries fearing its serious side effects [45].

In the current study, only 3.0% of the salmonella isolates were MDR, but none of the Shigella isolates were MDR, which was incomparable with the previous finding (12%) in South Africa [46].

Among the factors assessed in this study, accessibility of fruits to flies and dust was statistically significant with the occurrence of *Salmonella* species and S*higella* species in the juice houses. This is in agreement with an earlier finding inEthiopia; Addis Ababa [47]. The reason may be due to the fact that flies and dust may carry microorganisms from the environment and contaminate foods [27].

## Conclusion

In the present study, the overall prevalence of Salmonella and Shigella was found to be high. The high prevalence of isolates in this study revealed that potential hazard of fresh salads and fruit juices, and serious public health concern in the study area. These bacteria were highly resistant against ampicillin, tetracycline and amoxicillin-clavulanic acid. Where as ciprofloxacin, ceftriaxon, gentamicin, cotrimoxazole, and cholramphenicole were effective antibiotics. Accessibility of fruits to flies and dust was statistically associated with the occurrence of Salmonella and Shigella. Therefore, routine monitoring of juice houses should be promoted and regular evaluation of bacterial resistance pattern should be done for selective antimicrobial therapy. Furthermore, consistent training of juice makers on food safety and hygiene should be implemented by the concerned body.

## Data Availability

The data sets used and analyzed in this study are available from the principal author on reasonable request.

## Abbreviations

ATCC: American Type Culture Collection
BGI Ethiopia: Brasseries et Glaciers Internationals
CLSI: Clinical and Laboratory Standards Institute
CSA: Central Statistics Authority
EMB: Eosin Methylene Blue
FCC: Fecal Coliform Count
FDA: Food and Drug Administration
HE: Hekton Enteric Agar
MPN: Most Probable Number
SOP: Standard Operating Procedures
SPSS: Statistical Package for Social Science
TSI: Triple Sugar Iron Agar
UK: United Kingdom
WHO: World Health Organization
